# Designing a Coupling Agent with Aliphatic Polyether Chain and Exploring Its Effect on Silica/Natural Rubber Nanocomposites under the Action of Non-Rubber Contents

**DOI:** 10.3390/polym15030674

**Published:** 2023-01-28

**Authors:** Xiaobo Zhai, Xin Chen, Fangyuan Zheng, Dongli Han, Junchi Zheng, Xin Ye, Xiaolin Li, Liqun Zhang

**Affiliations:** 1 State Key Laboratory of Organic-Inorganic Composites, Beijing University of Chemical Technology, Beisanhuan East Road, Beijing 100029, China; 2 Yanshan Branch, National Engineering Research Center for Synthesis of Novel Rubber and Plastic Materials, Sinopec Beijing Research Institute of Chemical Industry, Beijing 102500, China; 3 Engineering Research Center of Elastomer Materials on Energy Conservation and Resources, Ministry of Education, Beijing University of Chemical Technology, Beijing 100029, China

**Keywords:** NR, coupling agent, VOC, NRCs

## Abstract

In order to prepare engineering tires with lower rolling resistance and better wet slip resistance in a more environmentally friendly way. In this study, a series of low volatile organic compound (VOC) M_x_–Si69 coupling agents (x = 1, 2, 3, 4, 5, 6, which means the number of ethoxy group in bis-(γ-triethoxysilylpropyl)-tetrasulfide (Si69) substituted by the aliphatic polyether chain) were applied to silica/NR nanocomposites to prepare tire tread with excellent performance. Firstly, M_1_–Si69 was substantiated as the best choice of M_x_–Si69 and Si69 to achieve comprehensive optima in the mechanical properties of silica/NR nanocomposites characterized by dynamic and static mechanical properties. Afterwards, the modification of silica with M_1_–Si69 induced by Non-Rubber Contents (NRCs) in silica/NR nanocomposites was revealed by comparing the filler network, micromorphology, and mechanical properties of isoprene rubber (IR) and NR nanocomposites. Furthermore, compared with Si69, the M_1_–Si69 coupling agent was found to conspicuously reduce the energy loss and improve the safety performance of engineering tires according to evaluations of the rolling resistance and dynamic thermomechanical properties of the silica/NR nanocomposites. Finally, the critical function of M_1_–Si69 in reducing ethanol (a kind of volatile organic compound (VOC)) emissions from the reaction of coupling agent and silica was disclosed by gas chromatography–mass spectrometry.

## 1. Introduction

In recent years, with the increasing shortage of petrochemical resources, reducing the energy loss of vehicles has received more and more attention [1,2]. The rolling resistance of tires, which is affected by the hysteresis effect of rubber nanocomposites, is an important component of the energy loss of a vehicle [3,4]. Therefore, reducing the hysteresis effect of rubber nanocomposites and improving the dynamic performance of tires have attracted the attention of researchers. In previous studies, “green tires” prepared by the nanocomposites of silica-reinforced solutions of polymerized styrene–butadiene rubber (SSBR) and butadiene rubber (BR) were proven to have extremely low rolling resistance values [5,6]. Road measurement results showed that a 22~35% rolling resistance reduction and 3~8% of fuel saving could be achieved with “green tires” compared with conventional tires [7,8]. In addition, according to Mikhaylov’s research, the development of “green tire” technology is also in line with the trend of social and economic development in the future [9,10].

Due to its insufficient tear and tensile strength, silica/BR/SSBR nanocomposites can only be used as the tread of a “green tire” for passenger vehicles [11,12]. However, in the field of engineering vehicles and trucks, natural rubber (NR) nanocomposites are still irreplaceable, and the demand to reduce rolling resistance to save the fuel consumption is also urgent the for tires used in these fields. Moreover, NR is an important strategic resource, derived from the latex of Hevea brasiliensis, that accounts for more than 30% of the world’s consumption of rubber hydrocarbons [13,14]. Because of its excellent mechanical properties, about 70% of produced NR is used in the tire industry, especially in engineering tires [15].

**Figure 1:**
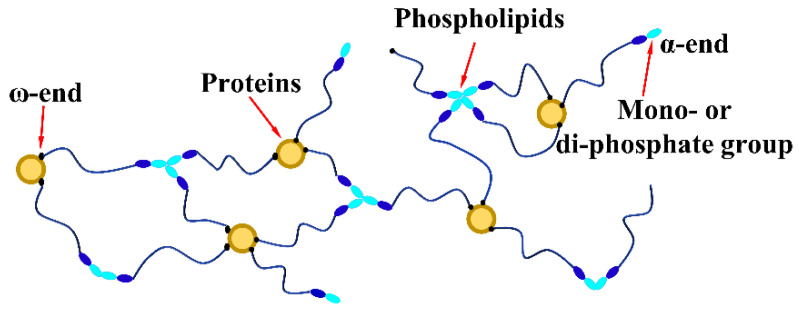
Molecular chain structure of NR [28].

In preparing tires for engineering vehicles and trucks, a certain amount of filler must also be added to the NR matrix to improve mechanical strength properties and reduce costs [16,17]. Silica is also an ideal filler for NR, as it can reduce the hysteresis effect of the rubber nanocomposite to meet the need of engineering tires with low rolling resistance [18,19]. In our previous research, it was found that silica in rubber must be combined with coupling agents to exert its excellent reinforcing properties, because good silica dispersion and proper interfacial interaction in silica/rubber nanocomposites can be achieved [20,21]. In addition, the emission of ethanol (a kind of volatile organic compound (VOC)), generated by the reaction of silica and coupling agents and volatilized at high temperatures, was also considered by us. Therefore, sulfurized cardanol polyoxyethylene ether, epoxy-terminated polybutadiene, and bis-epoxypropyl polysulfide were designed to avoid the emission of ethanol in preparing silica/rubber nanocomposites [22,23,24]. In recent research, we designed a series of M_x_–Si69 coupling agents that had different numbers of chains consisting of aliphatic polyethers in place of ethoxy groups in bis-(γ-triethoxysilylpropyl)-tetrasulfide (Si69), thereby reducing the volatilized ethanol produced by the hydrolysis of ethoxy groups; it was also found that M_2_–Si69 was the best choice in M_x_–Si69 and Si69 for decreasing the hysteresis effect of silica/BR/SSBR nanocomposites [25]. In this research, we intended to select the best of a series of M_x_–Si69 materials using in silica/NR nanocomposites to improve their dynamic properties and decrease VOC emissions during preparation. However, the molecular chain of NR contains not only 1000~3000 linear cis-1,4 isoprene polymer units but also 3~5 wt% of phospholipids, proteins, fatty acids and other non-rubber contents (NRCs; see Figure 1). S. Salina Sarkawi and Tiwen Xu et al. found that there are hydrogen and covalent bonds between NRCs and silica, which suggests potential effects of NRCs on the mode of silica modified by M_x_–Si69 [26,27]. Because M_x_–Si69 not only chemically grafts on the silica surface via a condensation reaction between hydroxyl groups but also physically adsorbs with silica through a strong hydrogen bond with the hydroxyl groups of the silica. Therefore, the effects of M_x_–Si69 under the action of NRCs on the properties of silica/NR nanocomposites needed to be clearly revealed.

In this work, a series of M_x_–Si69 coupling agents were first applied to silica/NR nanocomposites, and their cross-linking density, filler network, dynamic properties, and static properties were studied. Then, several silica/NR nanocomposites and silica/isoprene rubber (IR) nanocomposites were compared to study the differences between M_x_–Si69 and Si69 on silica modification under the action of NRCs. The mechanism of M_x_–Si69 coupling agent-modified silica in NR matrixes was proposed by comparing the differences between Si69 and M_x_–Si69 applied to silica/NR nanocomposites. Overall, in this research, a “green tire” with extremely low energy loss and ultra-high wet skid resistance was prepared, and the modification mechanism of M_x_–Si69 in NR matrixes was also revealed.

## 2. Experimental

### 2.1. Materials

The NR was provided by Hainan Natural Rubber Co., Ltd. (Haikou, Hainan, China); the IR was purchased from Zeon Co., Ltd. (Takaoka, Japan); and the silica was obtained from Degussa Co., Ltd. (Qingdao, Shandong, China). Si69 was purchased from Nanjing Youpu Chemical Co., Ltd. (Nanjing, Jiangsu, China), and M_x_–Si69 was synthesized by ourselves based on Si69. The accelerator was purchased from Kemai Chemical Co., Ltd. (Tianjin, China), and sulfur was purchased from Deli Chemical Co., Ltd. (Wuxi, Jiangsu, China); Zinc oxide and stearic acid were purchased from Lincheng Hongtu Technology Co., Ltd. (Xingtai, Hebei, China) and Fengyi Oil Technology Co., Ltd. (Lianyungang, Jiangsu, China), respectively.

### 2.2. Preparation of M_x_–Si69 Coupling Agent

The low VOC coupling agent M_x_–Si69 was obtained from a reaction between the terminal hydroxyl of fatty alcohol polyoxyethylene ether-9 (AEO-9) and the ethoxy group of coupling agent Si69. The reaction was carried out under the protection of nitrogen. AEO-9 and Si69 were first heated to 130 °C until no ethanol was produced in the reaction, and then the unreacted monomers were removed by vacuuming at 80 °C. By adjusting the molar ratio of AEO-9 to Si69 (1:1~6:1) and the dosage of tetrabutyl titanate of the catalyst (3 wt% of the total reactants), a series of low VOC coupling agents MxSi69 coupling agents containing different numbers of long alkyl chains of polyethers could be obtained.

### 2.3. Characteristics of Coupling Agents

**Figure 2:**
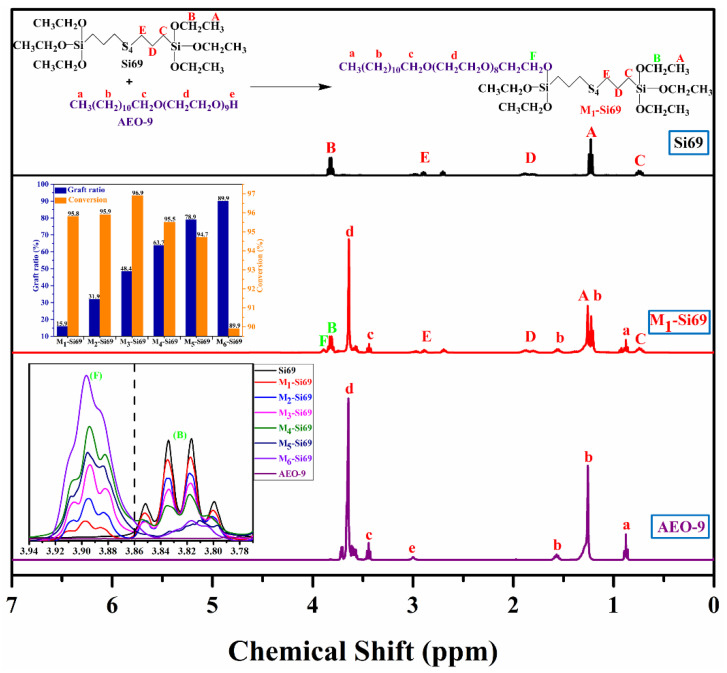
^1^H NMR spectra of Si69, M_x_–Si69, and AEO-9; graft ratio of Si69; and conversion of AEO-9 calculated with ^1^H NMR [25].

The structures and ^1^H NMR spectra of Si69 and M_x_–Si69 (take M_1_–Si69 as an example; x = 1, 2, 3, 4, 5, 6, which means the number of ethoxy group substituted by chains consisting of aliphatic polyethers) are shown in Figure 2. As previously reported [25], with increases in the aliphatic polyether ratio reacting with Si69, the area of F peak gradually increases and the area of B peak gradually decreases from the original 3.79–3.86 ppm, indicating that the ethoxy group has been replaced by a chain consisting of aliphatic polyethers. The reacting ratio is calculated according to ^1^H NMR data. The graft rate is defined as the ratio of AEO-9 participating in the reaction to the ethoxy group in Si69, and the conversion rate is defined as the ratio of AEO-9 participating in the reaction calculated from the ^1^H NMR spectrum to the amount of AEO-9 added during the reaction. Taking M_1_–Si69 as an example, if the conversion rate of AEO-9 was 100%, the graft rate of Si69 was 16.7%, as shown in Figure 2, and the conversion of aliphatic polyether in M_1_–Si69 ~M_6_–Si69 was about 90%, which demonstrated a high reaction efficiency.

### 2.4. Preparation of Rubber Nanocomposites

The formulation of the rubber nanocomposites is shown in Table 1. Silica/rubber nanocompounds were obtained through conventional in situ blending. First, plasticized rubber (NR or IR), silica, and coupling agent (Si69 or M_x_–Si69) were mixed together in an internal mixer, which has a chamber temperature of 55 °C and a rotational speed of 50 rpm, for 5 min. Then, an activator and an antioxidant were put into the chamber. After 2 min, a preliminary silica/rubber mixture was obtained. Second, the chamber temperature was adjusted to 150 °C and the rotor speed was adjusted to 30 rpm. All of the preliminary silica/rubber mixtures were kneaded for another 5 min to facilitate the reaction between the coupling agent and silica in the rubber matrix. After this process, all of the silica/rubber mixtures were cooled to room temperature. Third, the accelerator and a curing agent were uniformly blended in sequence with the cooled compound in a 6-inch mill at room temperature. The total mixing time was no more than 15 min.

The vulcanization of rubber nanocomposites was carried out on a flat vulcanizing machine. The vulcanization pressure was 15 MPa, and the vulcanization time was set according to the t_90_ obtained by a disc Vulkameter. The vulcanization time of the 2 mm sheet used to test tensile strength was (t_90_ + 3) min, the vulcanization time of the cylinder used to test heat build-up was 2 times (t_90_ + 3) min, and the vulcanization time of the solid tire used to test rolling resistance was 3 times (t_90_ + 3) min. These silica/rubber nanocomposites were denoted according to their components as NR–PS (pure silica), IR–PS, NR–8% Si69 (4.0 Si69; the dosage of the coupling agent was 8 wt% of the silica), NR–10% Si69 (5.0 Si69), IR–Si69, NR–M_x_–Si69, and IR–M_x_–Si69.

### 2.5. Characterization

Bound rubber content is often used to characterize interactions between a filler and rubber. Firstly, about 0.5 g (W_1_) of unvulcanized rubber composites was cut into 1 mm^3^ particles and wrapped into a copper mesh (the copper mesh mass is denoted as W_2_). Then, the sample was immersed in toluene for 72 h and soaked in a new toluene solution for another 24 h. Finally, the sample was placed in a 70 °C vacuum oven to a constant weight (W_3_). The bound rubber content of the sample was calculated with the following formula:

$$\mathrm{Bound}\;\mathrm{rubber}\;\mathrm{content}=\frac{W_{3}{-W}_{2}{-W}_{1}{\times \varepsilon }_{1}}{W_{1}{\times \varepsilon }_{2}}$$

where is ε_1_ was the mass fraction of filler and ε_2_ is the mass fraction of rubber.

The cross−linking density of the rubber nanocomposites was characterized by equilibrium swelling and calculated with the Flory–Rehner equation. A vulcanized sample (mass m_0_) was soaked in a toluene solution for 72 h, and then the toluene solution adsorbed onto the sample surface was removed with filter paper. Then, the sample was weighed to determine its weight (m_1_) as soon as possible. Finally, the sample was placed in a vacuum drying oven at 70 °C to a constant weight (m_2_), and the crosslinking density of the sample was calculated with the following formula:

(1) $$M_{c}=\frac{{-\rho }_{p}V_{s}V_{r}^{1/3}}{\ln(1-V_{r}){+V}_{r}{+\chi V}_{r}{}^{2}}$$

(2) $$V_{r}=\frac{{(m}_{2}{-m}_{f}{)/\rho }_{p}}{{(m}_{2}{-m}_{f}{)/\rho }_{p}{+(m}_{1}{-m}_{2}{)/\rho }_{s}}$$

(3) $$V_{c}=\frac{1}{{2M}_{c}}$$

where M_c_ is the molecular weight between molecular crosslinking points, m_f_ is the mass of filler in the test sample, ρ_p_ is the density of the polymer, V_s_ is the molar volume of toluene (106.4 cm^3^/mol), V_r_ is the volume fraction of rubber after swelling, ρ_s_ is the density of toluene (0.866 g/cm^3^), and V_c_ is the relative crosslinking density. The interaction parameters (χ) of rubber network and toluene was calculated as 0.393.

The Mooney viscosity of rubber was tested with a rotorless Mooney viscometer (Gotech Co., Ltd. Guangzhou, Guangdong, China) according to international standard ISO 289. The rubber was preheated at 100 °C for one minute and then tested for four minutes to obtain the corresponding Mooney viscosity value.

Hydrogen nuclear magnetic resonance (^1^H NMR) tests of the Si69, AEO-9 and M_x_–Si69 coupling agents were conducted on an AVANCE III 400 MHz NMR spectrometer (Bruker, Daltonik GmbH Co., Ltd. New York, NY, USA), for which, chloroform-d (CDCl_3_) was used as the solvent for testing.

The optimum vulcanization time of the rubber nanocomposites was determined with an M-3000A rotorless vulcanizer (Gotech Co., Ltd. Guangzhou, China) with a shear angle of 5° and a frequency of 200 cpm.

The filler network and hysteresis effect of nanocomposites were measured with an American Alpha Company RPA2000. The filler network of the unvulcanized rubber nanocomposites was tested at 60 °C, 1 Hz, and 0–200% strain. The vulcanizate was evaluated under conditions of 60 °C, 10 Hz, and 0~42% strain.

The filler dispersion and microstructure of the rubber nanocomposites were characterized and analyzed with an S-4800 scanning electron microscope from the Hitachi Company of Japan and an H-9500 transmission electron microscope from the JEOL Company of Japan.

Stress–strain curves were created with an AI-7000S1 electronic tensile machine (Gotech Co., Ltd. Guangzhou, Guangdong) according to international standard ISO 37. Samples were cut into dumbbell-shaped test strips with a length of 25 mm and tested at 500 mm/min.

Heat build-up characterization was conducted according to ISO 4666-3:2010 with an RH-2000N Heat Build-Up tester (Gotech Co., Ltd. Guangzhou, China). A cylindrical sample with a height of 25 mm was subjected to a compression test at 55 °C and a displacement of 2.225 for 30 min. Finally, the temperature difference at the bottom of the sample was recorded.

The energy loss of the rubber nanocomposites during rotation was determined with an RRS-II tire rolling resistance meter (Beijing Wanhui Technology Co., Ltd. Beijing, China). The rubber nanocomposites were set with a load of 30 kg and a rotating speed of 600 rpm.

The variation curve of the loss factor with temperature was characterized with a DMAVA-3000 dynamic thermomechanical analyzer of the French 01-dB company. The samples were tested with a deformation of 0.3% and a frequency of 10 Hz.

An Akron abrasion test was conducted with an MZ-4061 Akron abrasion meter (Jiangsu Mingzhu Testing Machinery Co., Ltd. Yangzhou, China). The samples were pre-ground for 800 revolutions and then ground for 3394 revolutions (equivalent to 1.61 km). The anti-wear properties of the rubber nanocomposites were evaluated via the volume difference between the samples before and after abrasion.

## 3. Results and Discussion

### 3.1. Properties of Silica/NR Nanocomposites Containing M_x_–Si69 

#### 3.1.1. Crosslink Density and Filler Network of Silica/NR Nanocomposites

**Figure 3:**
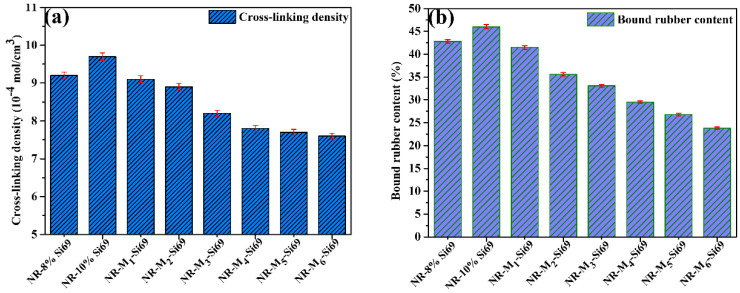
(**a**) Cross−linking density and (**b**) bound rubber content.

As shown in Figure 3a, NR–10% Si69 had the highest crosslink density of all silica/NR nanocomposites, and that of NR–8% Si69 was only lower than NR–10% Si69. With increases in the number of aliphatic polyether chain in M_x_–Si69, the cross-linking density of NR–M_x_–Si69 gradually decreased. As sulfur-containing coupling agents, Si69 and M_x_–Si69 chemically interacted with the silica surface through dehydration condensation during the process of mixing, and they reacted with rubber macromolecules via their active sulfur groups during the process of vulcanizing, resulting in the formation of a cross-linked structure in which multiple rubber molecules were bound to the same silica particle. Therefore, the cross-linking density of silica/NR nanocomposites is related to the actual number of coupling agent molecules used. The molecular weight of the aliphatic polyether chain in M_x_–Si69 was almost equal to that of Si69, which means that the actual number of M_x_–Si69 molecules significantly decreased with the increase in “x” at the same usage in these silica/NR nanocomposites, leading to a low crosslink density for NR–M_x_–Si69. Furthermore, the cross-linking density of NR–M_1_–Si69 was close to that of NR–8% Si69, although M_1_–Si69 had nearly twice the molecular weight of Si69, which means that M_1_–Si69 could more efficiently construct cross-linking networks in the rubber nanocomposites with fewer moles.

The bound rubber content and cross-linking density were used to characterize the interactions between the filler and rubber and the network structure of the silica/rubber nanocomposite. As shown in Figure 3b, with the increase in “x” in the series of Mx–Si69, the bound rubber content of the silica/NR composites showed a gradual downward trend. This can be explained by the gradual increase in physical interactions such as hydrogen bonds and van der Waals forces formed by the polyether long alkyl chain of M_x_–Si69 between the filler and rubber matrix and the decrease in the chemical bonding of the ethoxy and polysulfide groups.

**Figure 4:**
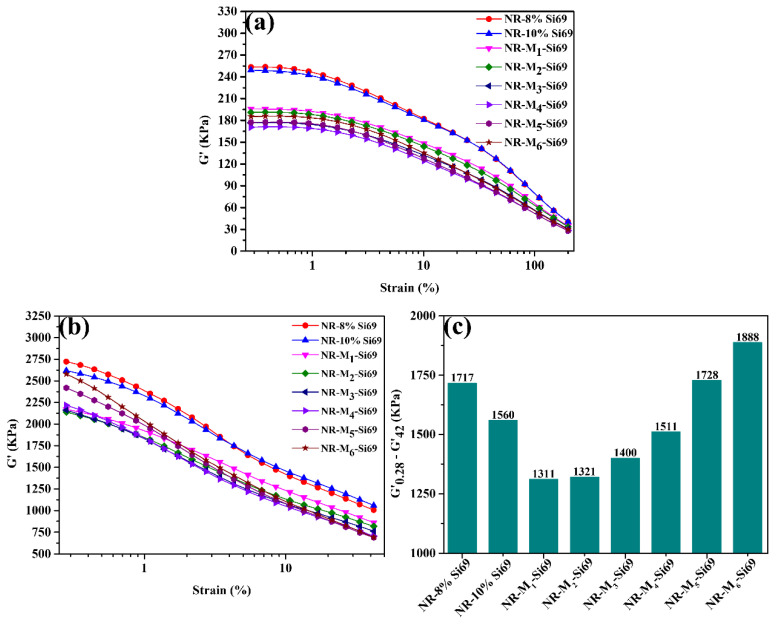
Storage modulus–strain curves of (**a**) unvulcanized silica/NR nanocomposites and (**b**) vulcanized silica/NR nanocomposites; (**c**) difference of storage moduli of vulcanizate silica/NR nanocomposites.

In the rubber nanocomposites, a rapidly decreased storage modulus with increasing strain is known as the Payne effect, which is closely related to the filler–filler network [29,30]. The Payne effect values of the studied unvulcanized rubber nanocomposites are exhibited in Figure 4a; the Payne effect values of NR–8% Si69 and NR–10% Si69 were almost the same and higher than that of NR–M_x_–Si69, indicating a weak filler–filler network in NR–M_x_–Si69. A curve of vulcanizate storage modulus versus strain is shown in Figure 4b, and the difference in the corresponding vulcanizate storage moduli is depicted in Figure 4c. NR–M_1_–Si69 showed the lowest Payne effect of the studied vulcanized rubber nanocomposites, which indicated that M_1_–Si69 could effectively weaken the filler network and reduce the agglomeration of nanoparticles (NPs) in the rubber matrix. As a simple chemical coupling agent, Si69 only reacted with one or two hydroxyl groups on the surface of silica. In contrast, the long polyether alkyl chain and ethoxy group were both present in the M_1_–Si69 coupling agent. During vulcanization, M_1_–Si69 could not only form chemical combinations with silanol groups through ethoxy groups but also cover a large number of unreacted silanol groups through its polyether structure. Therefore, a stable dispersion of silica with a weak filler–filler network in the rubber matrix was achieved by using M_1_–Si69.

#### 3.1.2. Static and Dynamic Properties of Silica/NR Nanocomposites

**Figure 5:**
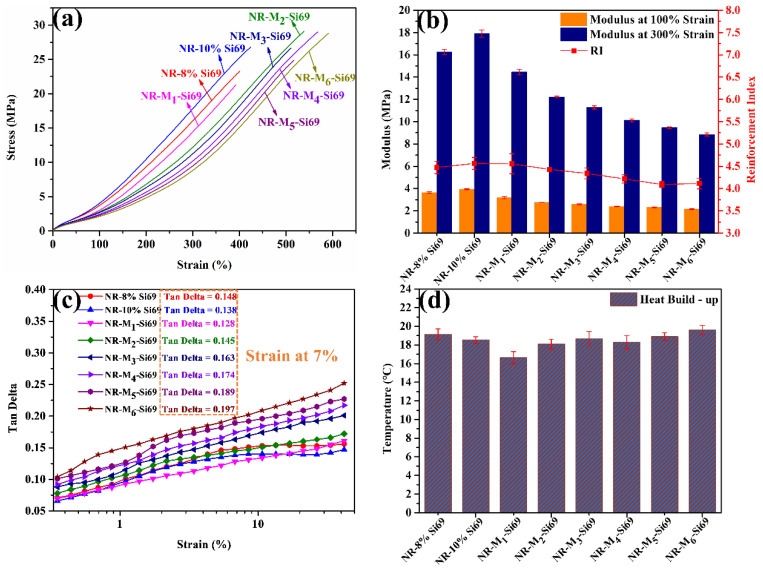
(**a**) Stress–strain curve, (**b**) modulus and reinforcement factor, (**c**) Tan delta at 60 °C, and (**d**) heat build-up of rubber nanocomposites.

The stress–strain curves of the silica/NR nanocomposites are shown in Figure 5a, and their corresponding moduli and reinforcement index (modulus at 300% divided by modulus at 100%) are depicted in Figure 5b. NR–8% Si69 and NR–10% Si69 showed higher moduli than NR–M_x_–Si69; meanwhile, the moduli of all NR–M_x_–Si69 materials decreased with increases in the number of aliphatic polyether chain in M_x_–Si69. The “coupling bridge” structure between silica and rubber, which was formed by Si69 or M_x_–Si69, played a significant role in improving the reinforcing efficiency of silica on rubber. NR–M_1_–Si69 had the highest reinforcement index of all silica/NR nanocomposites. The reinforcing efficiency was affected by both silica dispersion and the coupling structure existing between the silica and rubber. In this research, M_1_–Si69 was shown to be a proper combination for promoting silica dispersion and forming a coupling structure between silica and rubber.

Figure 5c shows the loss factor (Tan delta value at 60 °C) of the silica/NR nanocomposites, which is mainly used to reflect the energy loss caused by filler–filler and filler–rubber friction under a load with dynamic characteristics. In the tire industry, the loss factor at 7% strain is commonly used to characterize the rolling resistance of tires [31]. In theory, silica NPs that are adequately combined with rubber molecules under the premise of sufficient dispersion can weaken the friction in filler–filler and filler–rubber networks under dynamic conditions [32]. As shown in Figure 5c, NR–M_1_–Si69 had the lowest loss factor at 7% strain of all silica/NR nanocomposites, confirming that NR–M_1_–Si69 had the lowest rolling resistance. This can be explained by the facts that M_1_–Si69 effectively weakened the silica network in the rubber matrix and that the hysteresis loss caused by the destruction and reconstruction of the filler network was also reduced. In addition, NR–M_1_–Si69 had a higher bound rubber content and crosslinking density, which further reduced the energy loss caused by the friction between silica NPs and rubber molecular chains. Figure 5d shows the heat build-up of the silica/NR nanocomposites, which reflects the amount of energy loss due to the internal friction in the rubber nanocomposites under dynamic shock at constant pressure. NR–M_1_–Si69 also had the lowest heat build-up of all silica/NR nanocomposites, which aligned well with the loss factor measured with the RPA.

Ultimately, M_1_–Si69 is the best choice of the Si69 and series of M_x_–Si69 materials for achieving comprehensive optima in mechanical and dynamic properties. However, this is a different conclusion to that regarding the use of M_x_–Si69-series coupling agents in silica/SSBR nanocomposites. It can be deduced that the effect of the M_x_–Si69 coupling agent in silica/NR nanocomposites may be influenced by NRCs.

### 3.2. Mechanism of M_x_–Si69 on Silica Modification in NR Nanocomposites

In order to further study the effect of NRCs on the silica modification and use of M_x_–Si69 in silica/rubber nanocomposites, IR, which contains the same constituent units as NR but does not contain NRCs, was used for research. NR and IR additionally have similar molecular weights (as shown in Table 2) and raw rubber Mooney viscosity values (NR: 83; IR: 87).

#### 3.2.1. Cure Characteristics of Silica/NR Nanocomposites and Silica/IR Nanocomposites

**Figure 6:**
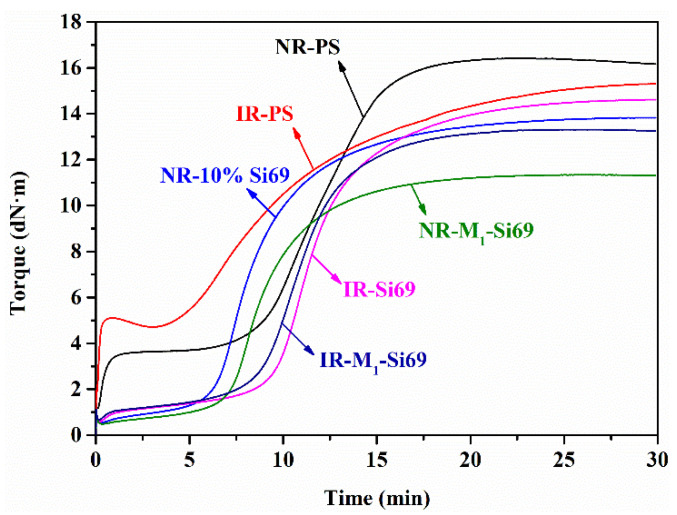
Vulcanization curves of NR and IR nanocomposites.

Vulcanization curves of the silica/NR nanocomposites and silica/IR nanocomposites are shown in Figure 6. As shown in Figure 6, the torque of NR–PS and IR–PS dramatically increased at the beginning of vulcanization due to the strong interaction between silica NPs that insufficiently dispersed in the rubber nanocomposites. The torque increase of NR–PS in the initial vulcanization stage was smaller than that of IR–PS, indicating that the interaction between silica NPs in NR–PS was weakened. It can be speculated that the NRCs, which were the only components in NR but not IR, play a role in weakening the interaction between silica NPs.

#### 3.2.2. Filler Network and Micromorphology of Silica/NR Nanocomposites and Silica/IR Nanocomposites

**Figure 7:**
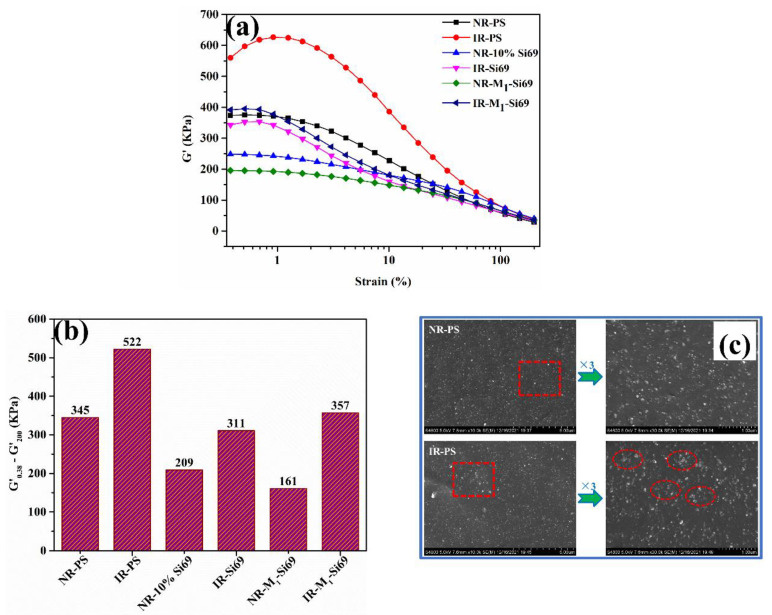
(**a**) Storage modulus versus strain, (**b**) difference of the storage modulus, and (**c**) micromorphology of the rubber nanocomposite.

The storage modulus versus strain curve of the rubber nanocomposites and the difference of the storage moduli are shown in Figure 7a,b. The difference of the storage modulus of IR–PS was 51.3% higher than that of NR–PS, indicating that the filler network of IR–PS was more developed than that of NR–PS. These results are consistent with the speculation described in Section 3.2.1. As mentioned by a previous researcher, proteins, which are a main component of NRCs, adsorb onto the silica surface by hydrogen bonds and van der Waals forces, thus hydrophobizing the silica surface and decreasing silica–silica interactions [26,27]. As shown in Figure 7c, the aggregate between silica NPs was more serious in vulcanized IR–PS than that in vulcanized NR–PS, which further proves the role of NRCs in aiding silica dispersion.

The Payne effect of IR–M_1_–Si69 was more obvious than that of IR–Si69; however, the Payne effect of NR–M_1_–Si69 was less obvious than that of NR–10% Si69. In our previous study, M_x_–Si69-series coupling agents also had no more significant effect on reducing the Payne effect than Si69 in silica/SSBR nanocomposites [25]. As coupling agents with ethoxy groups or aliphatic polyether chains at both ends, both Si69 and M_x_–Si69 have a chance to react with two different silica NPs. Therefore, silica NPs are isolated from each other by Si69 or M_x_–Si69, which is extremely effective in constraining the distance between silica NPs. The molecular number of Si69 is about twice that of M_1_–Si69 under the same mass usage, leading to more silica NPs being isolated in rubber nanocomposites that use Si69. The aliphatic polyether chain in M_x_–Si69 adsorbs onto the surface of silica via hydrogen bonds. Therefore, silica NPs are covered, which is also an effective in reducing the aggregation of silica. In the studied silica/IR nanocomposite, the isolation structure was found to be more effective than the coverage structure in weakening the interaction between silica NPs. In the studied silica/NR nanocomposite, NRCs, which are a component with a low molecular weight, could fully combine with silica in the form of hydrogen bonds under the premise of avoiding the influence of steric hindrance as much as possible. The hydrogen bond between NRCs and polyether had a chance to induce the aliphatic polyether chain in M_x_–Si69 to more fully cover silica NPs, thus strengthening the coverage structure in NR–M_1_–Si69.

**Figure 8:**
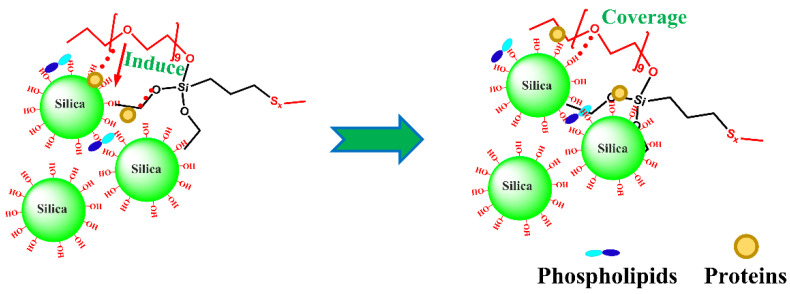
Schematic diagram of silica modified with M_x_–Si69 induced by NRCs.

In addition, the Payne effects of these six NR–M_x_–Si69 materials were almost the same, as shown in Figure 4. M_1_–Si69, with just one aliphatic polyether chain, could fully play the role of covering silica NPs when a large number of hydroxyl groups on the silica surface were occupied by NRCs in the form of hydrogen bonds, which was induced by NRCs to combine with silica. This is the reason why M_1_–Si69 was the best of the M_x_–Si69-series coupling agents at improving the silica/NR nanocomposites. A schematic diagram of the effect of NRCs on silica modified by M_x_–Si69 is shown in Figure 8.

**Figure 9:**
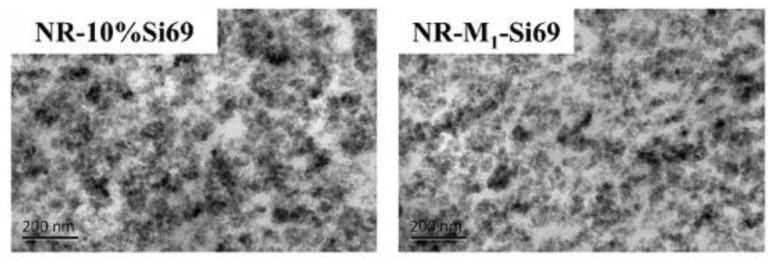
TEM images of NR–10% Si69 and NR–M_1_–Si69.

Figure 9 shows TEM images of vulcanized samples. NR–M_1_–Si69 had a more even silica dispersion than NR–10% Si69 because NR–10% Si69 contained large amounts of silica aggregates, as represented by dark area in Figure 9. During vulcanization, the polysulfide bond of Si69 or M_1_–Si69 broke and combined with the double bond of the rubber molecules. In this process, a “coupling bridge” was formed between silica NPs and rubber molecules, and the isolation structure between silica NPs was also destroyed. In contrast, the coverage structure formed by the aliphatic polyether chain in M_x_–Si69 for silica remained and played a role in preventing silica from re-aggregating during vulcanization.

#### 3.2.3. Static and Dynamic Performance of Silica/NR Nanocomposites and Silica/IR Nanocomposites

**Figure 10:**
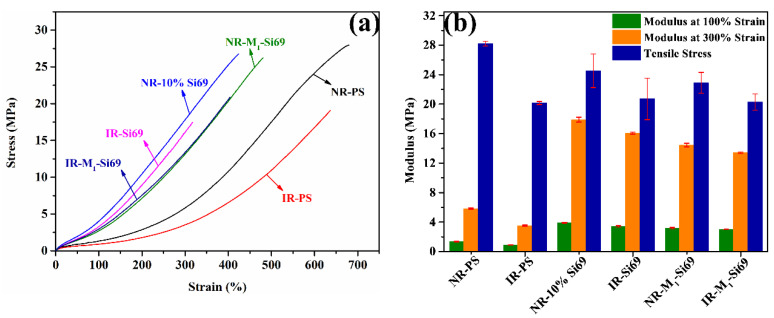
(**a**) Stress–strain curve; (**b**) modulus and tensile strength.

The static mechanical properties of the silica/NR nanocomposites and silica/IR nanocomposites are shown in Figure 10. NR–PS had a higher modulus than IR–PS, which was also due to the relatively good silica dispersion in NR–PS aided by NRCs. In addition, compared with IR–PS, NR–PS displayed a higher tensile strength, which was due to the NRCs adsorbing onto the surface of silica via hydrogen bonds. During the tensile test, rubber molecules slid across the filler surface with the help of the adsorbed NRCs, which endowed the silica/NR nanocomposites with a higher tensile strength [33].

**Figure 11:**
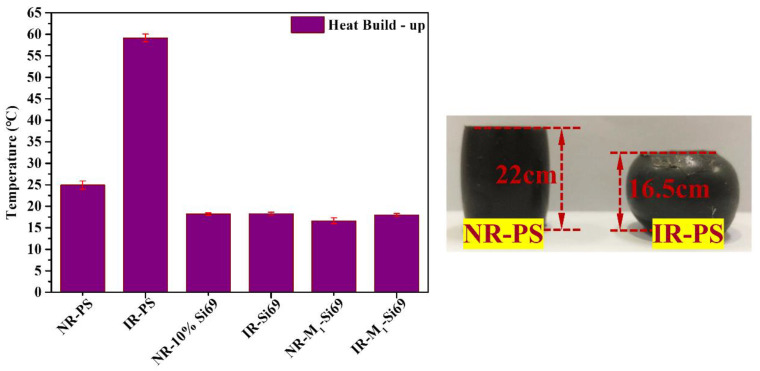
Heat build-up of the silica/rubber nanocomposites.

The dynamic performance of the silica/rubber nanocomposites is displayed in Figure 11. IR–PS exhibited extremely high heat build-up values and severe deformation, which was due to the serious aggregation of silica. NR–PS had a lower heat build-up value than IR–PS because NRCs improved its silica dispersion. NR–M_1_–Si69 had the lowest heat build-up values of all six silica/rubber nanocomposites, which proves that the coverage structure for silica was formed by the aliphatic polyether chain in M_x_–Si69 induced by NRCs and that can improve the dynamic properties of silica/rubber nanocomposites.

In this research, the effect of NRCs on the use of M_x_–Si69 was analyzed by comparing the properties of silica/NR nanocomposites and silica/IR nanocomposites. NRCs were found to not only adsorb on the silica surface but also improve the efficiency of aliphatic polyether chains in improving the dispersion of silica. Therefore, in the silica/NR nanocomposites, M_1_–Si69, which only contained one aliphatic polyether chain, was sufficient to improve the dispersion of silica by covering hydroxyl groups on the silica surface. The sulfur components decreased with increases in the number of aliphatic polyether chains in M_x_–Si69 under the same dosage, which was detrimental to the cross-linking density of the silica/rubber nanocomposites. Of the M_x_–Si69-series coupling agents, M_1_–Si69 could balance the silica dispersion and cross-linking density of the nanocomposites better than others. Therefore, NR–M_1_–Si69 exhibited ideal performance.

#### 3.2.4. Rolling Resistance of the Silica/NR Nanocomposites

**Figure 12:**
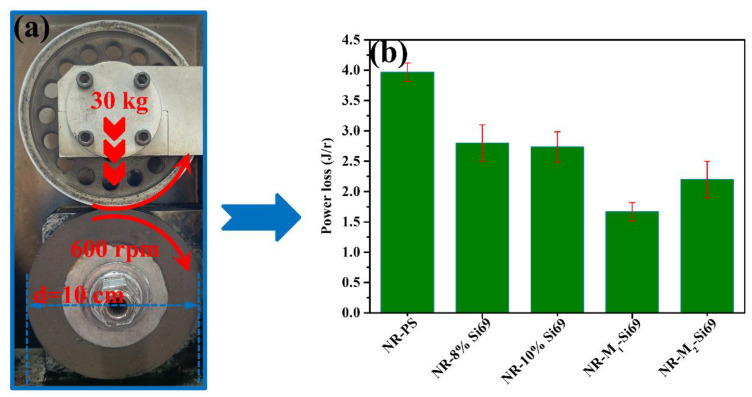
(**a**) Model of the tire energy loss test; (**b**) energy loss.

To further evaluate the energy loss of M_1_–Si69 applied to rubber nanocomposites for tires, five rubber nanocomposites containing different coupling agents were prepared as tire models for testing. As shown in Figure 12, the tires prepared with NR–8% Si69 and NR–10% Si69 had almost the same energy losses, indicating that the 8% Si69 used in the silica/NR nanocomposites was enough to reduce dynamic energy loss because the number of reactive hydroxyl groups on the silica surface was limited. Furthermore, the tire prepared with NR–M_1_–Si69 had a 39% lower energy loss than the tire prepared with NR–8% Si69. According to previous reports, 3% of fuel consumption can be saved when rolling resistance is dropped by 10%. If 100 million tons of fuel is consumed per year, a “green tire” made of NR–M_1_–Si69 instead of NR–8% Si69 could save about 2 million tons of fuel and 5 million tons of carbon dioxide [34]. The energy loss of tires mainly comes from the mutual friction between silica NPs under cyclic reversed loading [35]. M_1_–Si69 was shown to combine good silica dispersion and a sufficient “coupling bridge” between silica and rubber, which effectively weakens the mutual friction of silica NPs.

#### 3.2.5. Wet-Skid Resistance of the Silica/NR Nanocomposites

**Figure 13:**
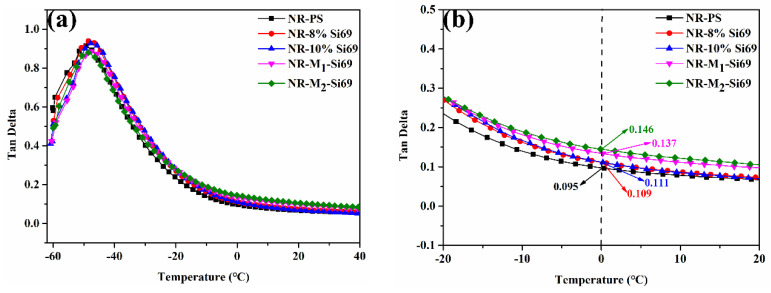
DMTA curves of silica/rubber nanocomposites.

In the tire industry, a 0 °C loss factor is commonly used to evaluate the wet skid resistance of tires [36,37]. To study the safety performance of tires on wet-skid roads, the DMTA testing of five nanocomposites was conducted. As shown in Figure 13, NR–PS had the lowest 0 °C loss factor, and NR–M_1_–Si69 and NR–M_2_–Si69 had higher 0 °C loss factors than NR–10% Si69 and NR–8% Si69. This was because the silica NPs showed good dispersion in the vulcanized rubber nanocomposites due to the help of the coverage structure formed by the aliphatic polyether chain in M_x_–Si69, leading more rubber molecular chains in NR–M_1_–Si69 and NR–M_2_–Si69 participating in the relaxation motion at 0 °C. The wet skid resistance of NR–M_1_–Si69 was improved by more than 25% compared with NR–8% Si69, which significantly improved the safety performance of the “green tires”.

#### 3.2.6. Akron Abrasion Loss of the Silica/NR Nanocomposites

**Figure 14:**
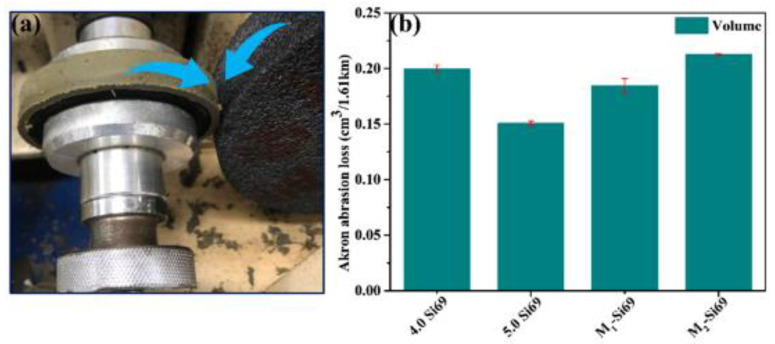
(**a**) Model of Akron wear test; (**b**) Akron wear volume.

**Figure 15:**
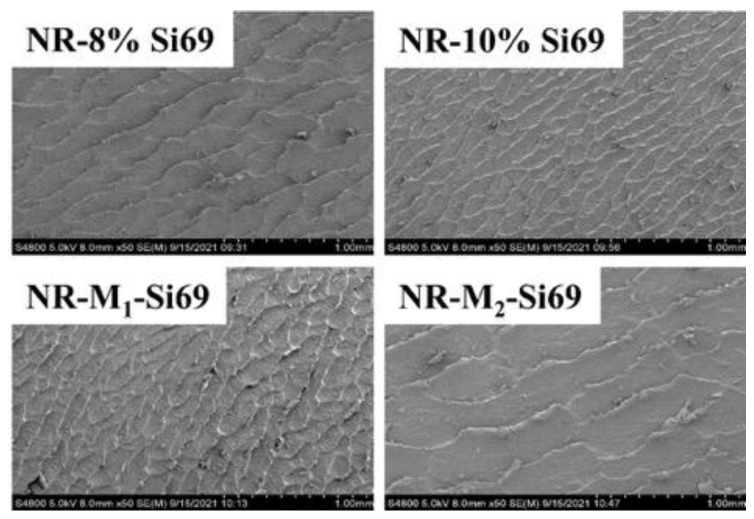
SEM images of Akron wear surface.

The wear resistance of tires is affected by multiple factors such as filler dispersion, interfacial bonding, and cross-linking density [38,39,40]. Figure 14 shows a model of the Akron abrasion measurement and the Akron wear volume. NR–10% Si69 had the least wear volume because of its high cross-link density. NR–M_1_–Si69 and NR–8% Si69 had similar relative cross-linking densities, but NR–M_1_–Si69 had less wear volume and narrower wear stripes (Figure 15) than NR–8% Si69, meaning that the wear resistance of the silica/NR nanocomposite was improved by M_1_–Si69 under the same cross-linking density.

#### 3.2.7. VOC Contents of Different Coupling Agents Reacted with Silica

**Figure 16:**
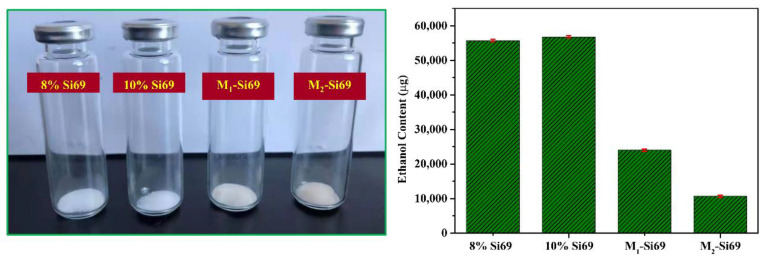
Characterization of the VOC content of coupling agent-modified silica.

The VOC content (ethanol) generated by the reaction of the coupling agent with silica was characterized with gas chromatography–mass spectrometry. As shown in Figure 16, the silica and coupling agent were evenly mixed and placed in a 20 mL sample bottle. These bottles were heated at 150 °C for 30 min, and then the VOC content generated by the reaction was tested. When the ratio of Si69 mixed with silica increased from 8% to 10%, the VOC content produced by the reaction showed no significant change, indicating that the reaction between Si69 and reactive hydroxyl groups on the silica surface was limited. However, when the silica was modified by M_1_–Si69 and M_2_–Si69, VOC emissions were reduced by 57% and 80%, respectively, which demonstrates the importance of replacing Si69 with M_1_–Si69 in reducing VOC emissions during the preparation of rubber nanocomposites.

## 4. Conclusions

In conclusion, NRCs can beneficially weaken silica networks and improve the dispersion of silica in rubber matrixes. In addition, the combination of silica and polyether segments in M_x_–Si69 that is induced by NRCs was found to improve the dispersion of silica in NR matrixes due to a more complete coverage structure for silica. Therefore, M_1_–Si69, which only has one aliphatic polyether chain, can provide enough coverage structure for silica in silica/NR nanocomposites. Moreover, M_1_–Si69 was found to be more efficient than Si69 in improving the dynamic properties of silica/NR nanocomposites. In this research, compared with NR–8% Si69, NR–M_1_–Si69 saved about 39% of energy loss and improved tire wet skid resistance by more than 25%. In addition, compared with Si69, the M_1_–Si69 coupling agent reduced VOC (ethanol) emissions by more than 50%. Therefore, the application of the newly designed M_1_–Si69 coupling agent in silica/NR nanocomposites can not only endow a tire with extremely low energy loss and ultra-high wet skid resistance but also significantly reduce VOC gas emissions, thus providing a new strategy for the manufacturing of high-performance truck tires from the perspective of reducing VOC gas emissions.

## Figures and Tables

**Table 1:** Formulation of silica/rubber nanocomposites.

Materials/Phr ^a^	NR–PS	IR–PS	NR–8% Si69	NR–10% Si69	IR–Si69	NR–M_x_–Si69	IR–M_x_–Si69
NR	100	0	100	100	0	100	0
IR		100	0	0	100	0	100
silica	50	50	50	50	50	50	50
M_x_–Si69	0	0	0	0	0	5	5
Si69	0	0	4	5	5	0	0
other additives ^b^	14	14	14	14	14	14	14

^a^ Parts per hundred of rubber. ^b^ Stearic acid 2.0 (activator), zinc oxide 5.0 (activator), N-1,3-dimethylbutyl-N′-phenyl-p-phenylenediamine 2.0 (antioxidant), N-cyclohexyl-2-benzothiazole sulfenamide 2.0 (accelerator), 1,3-diphenylguanidine 1.0 (accelerator), and sulfur 2.0 (curing agent).

**Table 2:** Molecular weight and distribution of NR and IR.

Sample	*M_n_* ^a^/(g/mol)	*M_w_* ^b^/(g/mol)	Ð ^c^
NR	488,346	1,269,122	2.60
IR	484,641	1,157,461	2.39

^a^ *M_n_* refers to number-average molecular weight. ^b^ *M_w_* refers to weight-average molecular weight. ^c^ refers to polydispersity index.
